# Supplementary material for: MITF, TFEB, and TFE3 drive distinct adaptive gene expression programs and immune infiltration in melanoma
Source: Cell Rep. Author manuscript; Available in PMC 2026 Jan 23. (PMC12828906; doi:10.1016/j.celrep.2025.116499)
Supplement: 1 [file NIHMS2132725-supplement-1.pdf]

Cell Reports, Volume *44*

Supplemental information

MITF, TFEB, and TFE3 drive distinct adaptive gene expression programs and immune infiltration in melanoma

Diogo Dias, Erica Oliveira, Román Martí-Díaz, Sarah Andrews, Ana Chocarro-Calvo, Alice Bellini, Laura Mosteo, Yurena Vivas García, Jagat Chauhan, Linxin Li, José Manuel García-Martinez, José Neptuno Rodriguez-López, Silvya Stuchi Maria-Engler, Colin Kenny, Javier Martínez-Useros, Custodia García-Jiménez, Luis Sanchez-del-Campo, Pakavarin Louphrasitthiphol, and Colin R. Goding

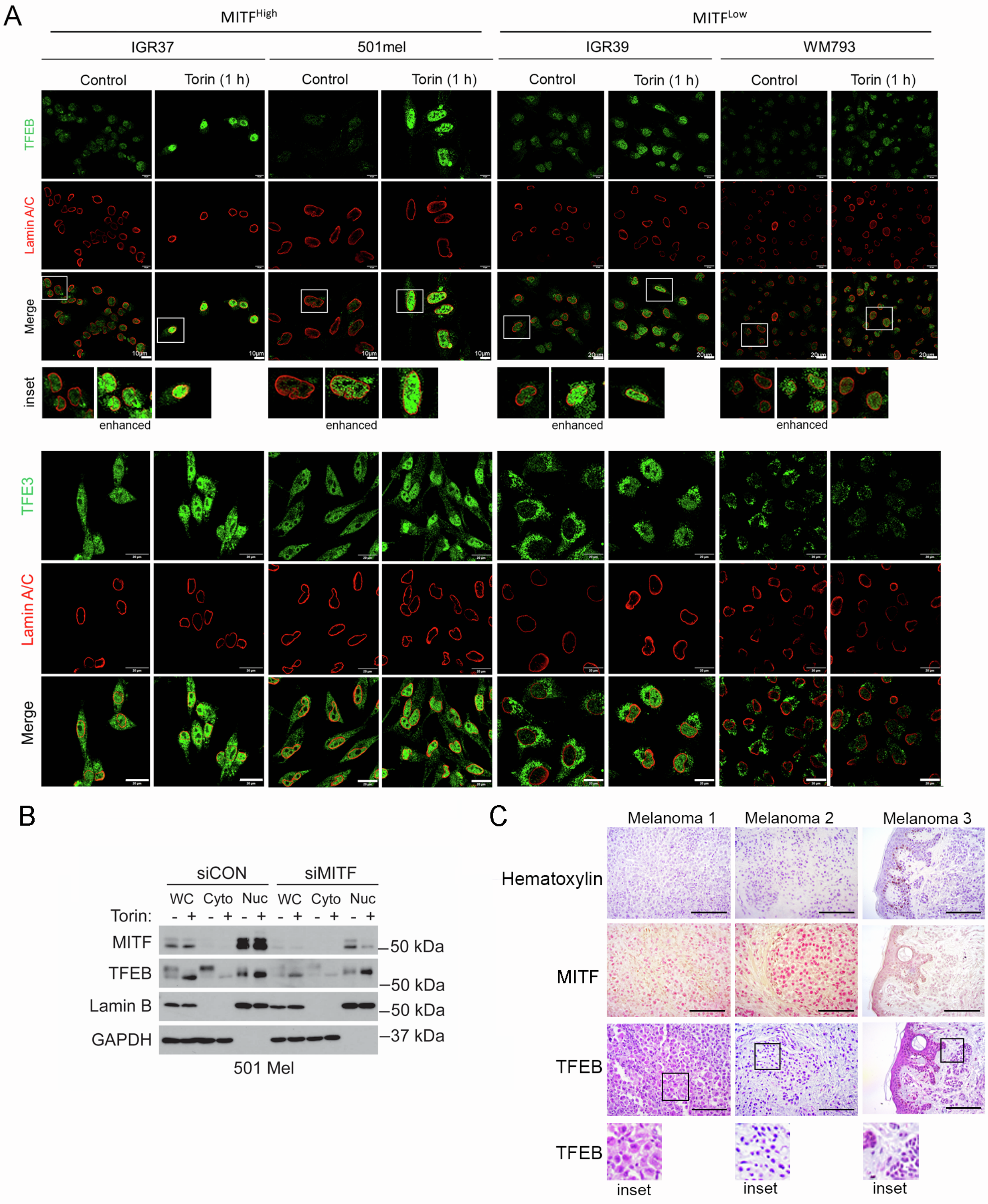

Figure S1 related to Figure 1. Predominantly nuclear localization of TFEB in melanoma cells
(A) Immunofluorescence in indicated melanoma cell lines treated or not with 250 nM Torin for 1 h using anti-TFEB (green; top panels), anti-TFE3 (green; bottom panels) and anti-Lamin B (red). Scale bars = 10 μm for 501mel and IGR37, and 20 μm for the IGR39 and WM793 cells for the TFEB panels and 20 μm for the TFE3 panels. Insets for TFEB panels show magnified regions below, and for the control samples an enhanced image is shown to enable to nuclear localization of TFEB to be readily visualized.
(B) Western blot showing fractionated 501mel cell extracts using Lamin B and GAPDH as markers for the nuclear and cytoplasmic fractions. Cells were transfected with control or siMITF for 48 h before treatment with 250 nM Torin or DMSO before fractionation.
(C) Immunohistochemistry on three human melanomas using anti-MITF or anti-TFEB antibodies (lower panels) and hematoxylin staining (upper panels). Scale bars = 50 μm.

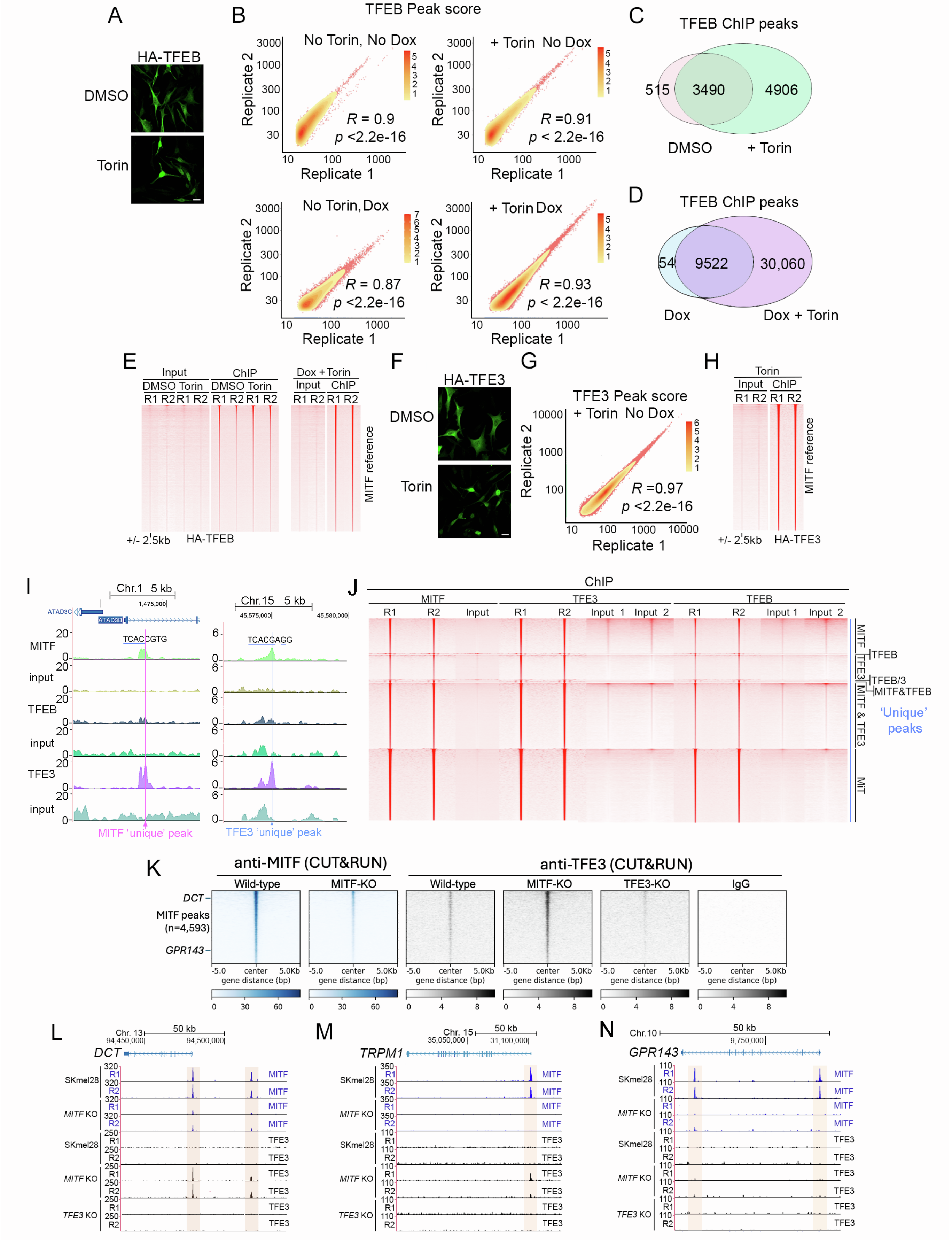

Dias et al Supplemental Figure S2

Figure S2 related to Figure 3. ChIP-seq of HA-TFEB and HA-TFE3.
(A) Immunofluorescence using anti-HA antibody showing nuclear accumulation of HA-TFEB after treatment with 250 nM Torin for 6 h. Scale bar = 10 μm.
(B) Inter-replicate correlation of ChIP-seq of HA-TFEB performed under indicated conditions. R = Spearman rho.
(C-D) Venn Diagrams showing significant HA-TFEB ChIP-peaks from 501mel cells with or without Torin treatment (C) or in the presence of 5 ng Doxycycline with or without 250 nM Torin (D).
(E) Heatmap of tag density of each HA-TFEB ChIP-seq experiment using 0 or 5 ng doxycycline with or without 250 nM Torin as indicated using ranked MITF ChIP-seq narrow peak as reference genome coordinate and extending +/- 2.5kb.
(F) Immunofluorescence using anti-HA antibody showing nuclear accumulation of HA-TFE3 after treatment with 250 nM Torin for 6 h. Scale bar = 10 μm.
(G) Inter-replicate correlation of ChIP-seq of HA-TFE3 performed under indicated conditions.
(H) Heatmap showing tag density of each HA-TFE3 ChIP-seq experiment of HA-TFE3 expressed using 0 ng doxycycline with 250 nM Torin using the same ranked MITF narrow peaks reference as in E.
(I) UCSC genome browser screenshots showing ChIP profiles for input control and HA-TFEB, HA-TFE3 and HA-MITF at indicated loci called as an MITF 'unique' peak (left) and TFE3 'unique' peak (right). Only one replicate is shown. Similar results were obtained for replicate 2. Sequences related to the MITF family binding consensus are shown above the tracks with the consensus-related sequence underlined.
(J) Heatmap of tag density of each HA-MITF, HA-TFEB and HA-TFE3 ChIP-seq experiment with input controls (left to right) corresponding to 'unique peaks' called for each combination of factor as identified in Figure 3F.

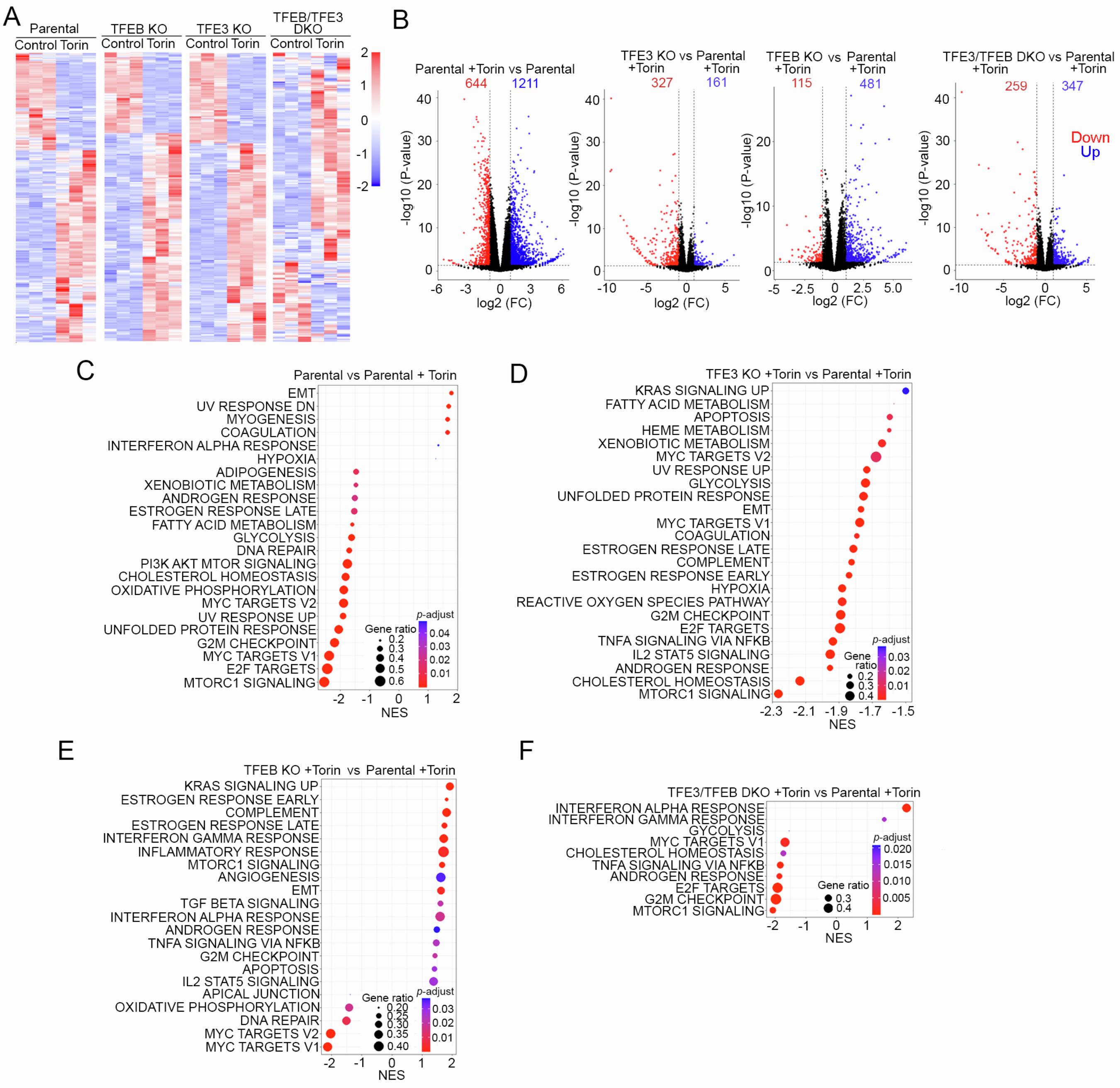

Figure S3 related to Figure 4. Differential regulation of genes in parental or TFEB/TFE3 KO 501mel cells after Torin treatment.

(A) Heatmaps based on triplicate RNA-seq showing differential gene expression of parental or indicated 501mel *TFEB* and *TFE3* KO cell lines before or after treatment with 250 nM Torin for 12 h.

(B) Volcano plots showing numbers of significantly (FC ≥ 2, p = <0.05) differentially expressed genes comparing parental cells and indicated 501mel KOs in the presence or absence of 250 nM Torin for 12 h as indicated.

(C-F) GSEA plots showing significantly differentially enriched gene sets comparing parental or indicated 501mel *TFEB* and *TFE3* KO cells treated or not with 250 nM Torin for 12 h, as indicated.

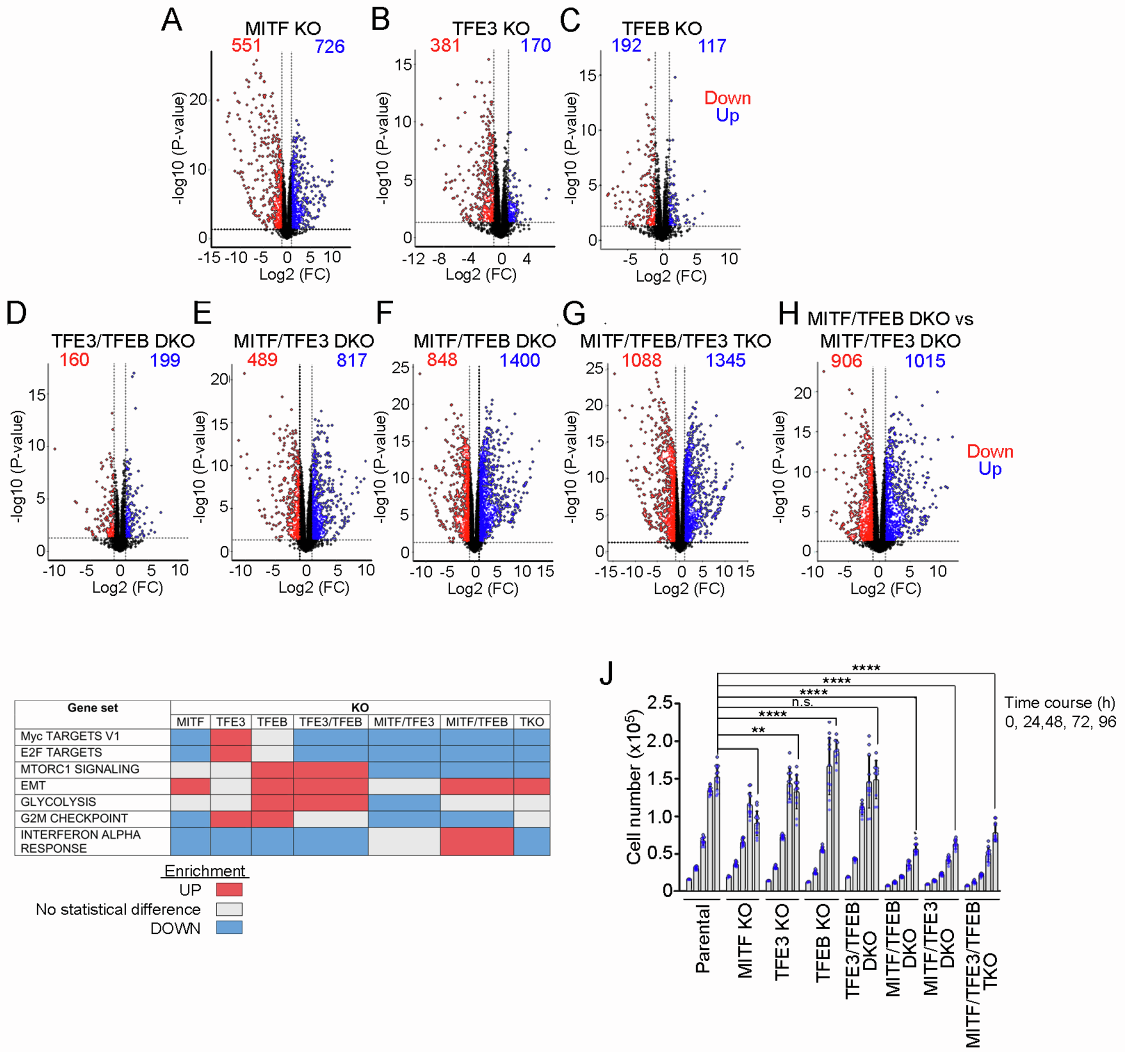

Figure S4 related to Figure 5. MITF/TFE factors control proliferation.

(A-H) Volcano plots showing numbers of significantly (FC ≥ 2, p = <0.05) differentially expressed genes comparing parental cells and indicated B16-F10 KO cells.

(I) Summary of GSEA analyses presented in Figure 5.

(J) CyQuant assays of parental or indicated *MITF*, *TFEB* and *TFE3* B16-F10 KO cells over time after plating of 4,000 cells at time =0 h. N=12 (3 biological replicates in quadruplicate). Error bars indicate S.D. **= p < 0.01; **** = p < 0.0001; *ns* = not significant when compared to WT cells at 96 h. Linear Mixed-Effects Models was used first to model the slopes then a post-hoc comparison using estimated marginal means accounting for two interacting factors (cell line and time) was used for pairwise comparison. The Bonferroni method was used to adjust p-values for multiple comparisons.

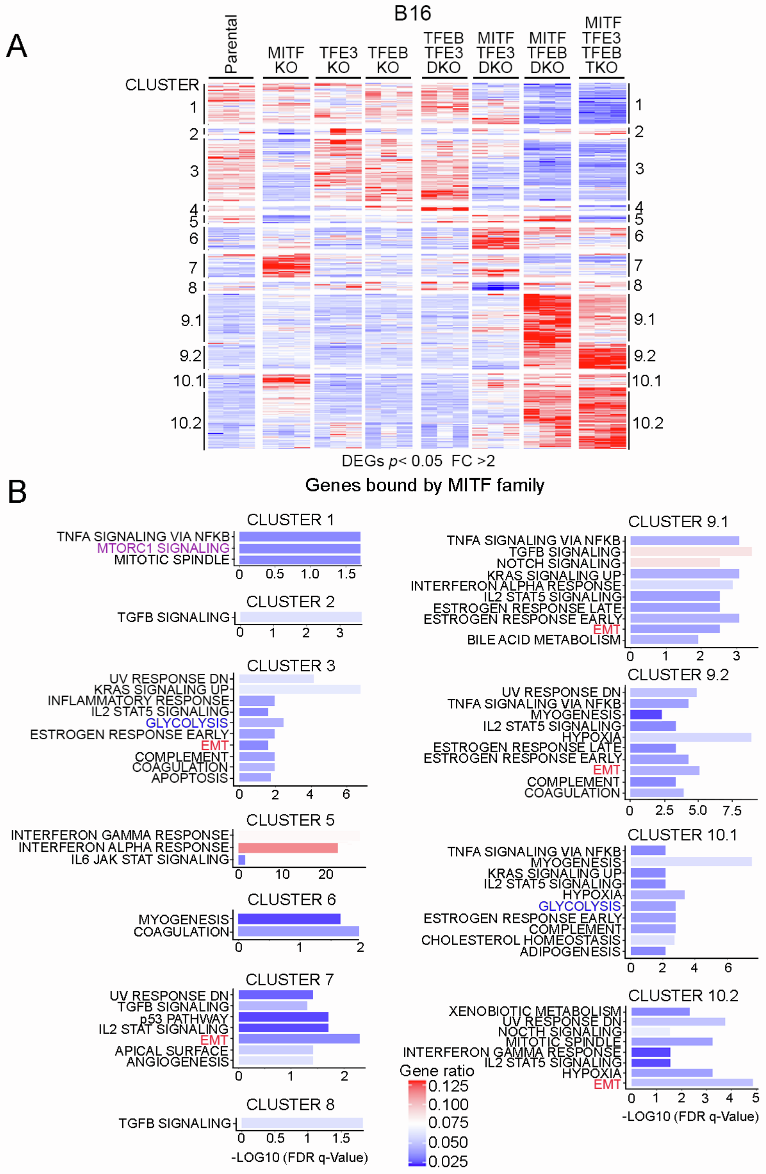

Figure S5 related to Figure 5. Differential regulation of genes bound by all MITF family members

(A) Heatmaps based on triplicate RNA-seq showing differential gene expression between parental B16-F10 cells and indicated *MITF*, *TFEB* and *TFE3* KO cell lines. Only those genes commonly bound by all three factors (shown in Figure 3F) are used in the analysis.

(B) Enrichment of indicated gene sets within each cluster corresponding to those identified in panel (A)

Related to Table S3

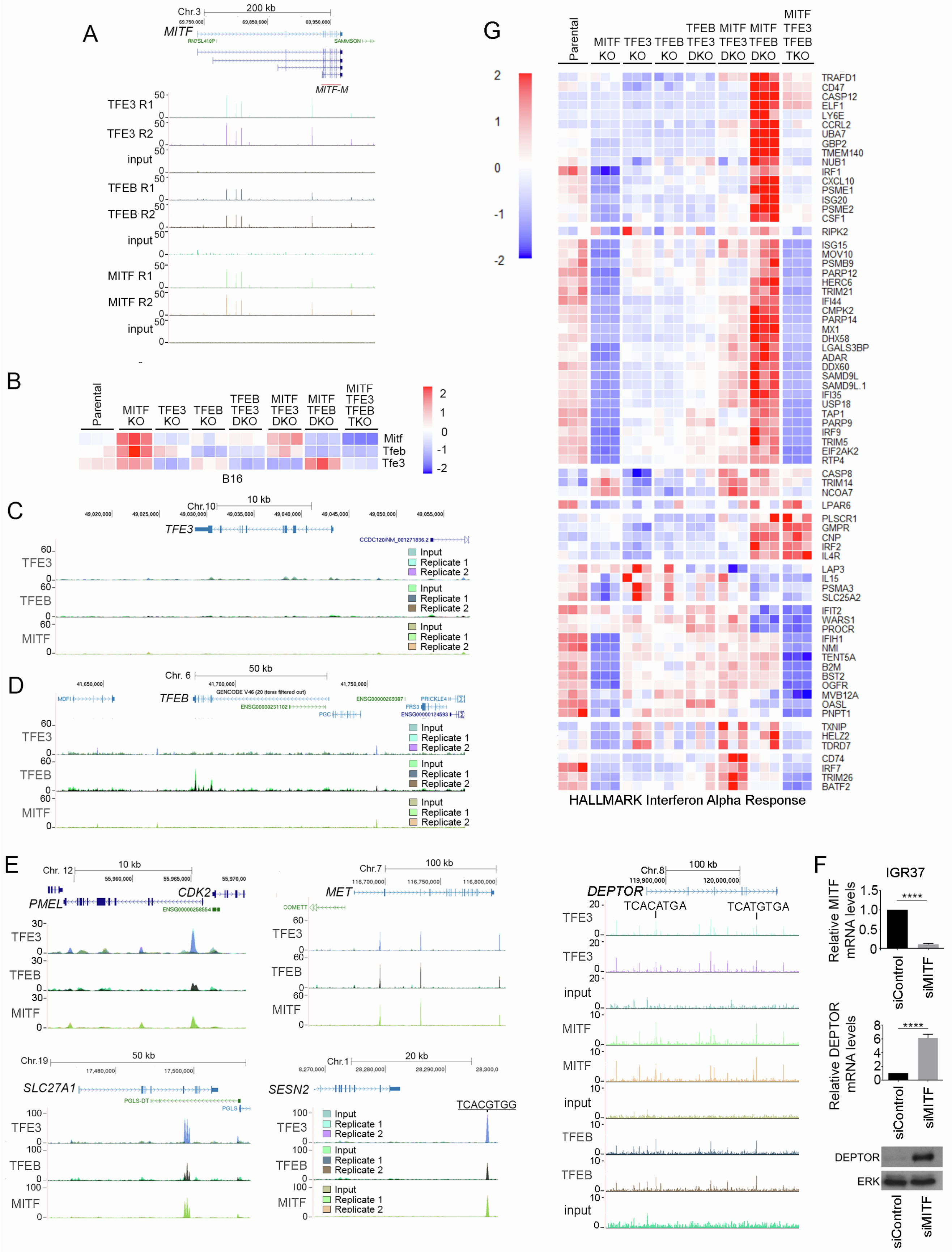
A
Chr.3
200 kb
MITF
MITF-M
TFE3 R1
TFE3 R2
input
TFEB R1
TFEB R2
input
MITF R1
MITF R2
input
B
Parental
MITF KO
TFE3 KO
TFEB KO
TFEB TFE3 DKO
MITF TFE3 DKO
MITF TFEB DKO
MITF TFE3 TFEB TKO
Mitf
Tfeb
Tfe3
B16
C
Chr.10
10 kb
TFE3
TFE3
TFEB
MITF
Input
Replicate 1
Replicate 2
D
Chr. 6
50 kb
TFEB
TFE3
TFEB
MITF
Input
Replicate 1
Replicate 2
E
Chr. 12
10 kb
PMEL
CDK2
TFE3
TFEB
MITF
Chr.7
100 kb
MET
TFE3
TFEB
MITF
Chr.19
50 kb
SLC27A1
TFE3
TFEB
MITF
Chr.1
20 kb
SESN2
TCACGTGG
TFE3
TFEB
MITF
Input
Replicate 1
Replicate 2
Chr.8
100 kb
DEPTOR
TCACATGA
TCATGTGA
TFE3
TFE3
input
MITF
MITF
input
TFEB
TFEB
input
F
IGR37
Relative MITF mRNA levels
****
siControl
siMITF
Relative DEPTOR mRNA levels
****
siControl
siMITF
DEPTOR
ERK
siControl
siMITF
G
Parental
MITF KO
TFE3 KO
TFEB KO
TFEB TFE3 DKO
MITF TFE3 DKO
MITF TFEB DKO
MITF TFE3 TFEB TKO
TRAFD1
CD47
CASP12
ELF1
LY6E
CCRL2
UBA7
GBP2
TMEM140
NUB1
IRF1
CXCL10
PSME1
ISG20
PSME2
CSF1
RIPK2
ISG15
MOV10
PSMB9
PARP12
HERC6
TRIM21
IFI44
CMPK2
PARP14
MX1
DHX58
LGALS3BP
ADAR
DDX60
SAMD9L
SAMD9L.1
IFI35
USP18
TAP1
PARP9
IRF9
TRIM5
EIF2AK2
RTP4
CASP8
TRIM14
NCOA7
LPAR6
PLSCR1
GMPR
CNP
IRF2
IL4R
LAP3
IL15
PSMA3
SLC25A2
IFIT2
WARS1
PROCR
IFIH1
NMI
TENT5A
B2M
BST2
OGFR
MVB12A
OASL
PNPT1
TXNIP
HELZ2
TDRD7
CD74
IRF7
TRIM26
BATF2
HALLMARK Interferon Alpha Response

Figure S6 related to Figure 6. Binding by MITF family members to key genes and regulation of the Interferon Alpha Response gene set.
(A) UCSC genome browser screenshots showing binding by MITF, TFEB and TFE3 at the *MITF* locus.
(B) Heatmap based on triplicate RNA-seq showing relative expression of *MITF*, *TFEB* and *TFE3* in indicated parental and B16-F10 KO cell lines.
(C-D) UCSC genome browser screenshots showing ChIP-seq profiles of HA-TFE3 and HA-TFEB and HA-MITF at the indicated loci. Binding by TFEB to the *TFEB* gene in panel (D) primarily represents an input signal.
(E) UCSC genome browser screenshots showing ChIP-seq profiles of HA-TFE3 and HA-TFEB and HA-MITF at the indicated loci.
(F) Results of quantitative RT PCR for *MITF* and *DEPTOR* mRNAs in IGR37 human melanoma cells transfected with control or MITF-specific siRNA. n=3. Error bars = S.D. **** = p < 0.0001. Lower panel shows the corresponding western blot.
(G) Heatmap showing relative gene expression based on triplicate RNA-seq of parental B16-F10 and derived *MITF*, *TFEB* and *TFE3* KO cells showing genes from the HALLMARK Interferon Alpha Response gene set.
